# Trusting your gut: a hairy situation—gastric trichobezoar case report

**DOI:** 10.3389/fped.2026.1748583

**Published:** 2026-02-26

**Authors:** Amy Yeung, Lauren Dankner, J. Antonio Quiros, Eric Lazar, Linda Li, Khadijah Walker, Sita Chokhavatia

**Affiliations:** 1 Department of Internal Medicine, The Valley Hospital, Paramus, NJ, United States; 2 Department of Pediatric Gastroenterology, Valley Medical Group, Paramus, NJ, United States; 3 Department of Pediatric General Surgery, Valley Medical Group, Paramus, NJ, United States; 4 Department of Gastroenterology, Valley Medical Group, Paramus, NJ, United States

**Keywords:** EGD (esophagogastroduodenoscopy), imaging, pediatric, trichobezoar, trichophagia, trichotillomania (hair pulling disorder)

## Abstract

Trichobezoars are rare masses made from ingested hair that are commonly seen in young females. We report a case of a 7-year-old girl who presented with generalized abdominal pain and rapid weight loss. Initial evaluation with radiography, magnetic resonance imaging (MRI), and upper gastrointestinal series (UGI) suggested superior mesenteric artery (SMA) syndrome but failed to provide a definitive diagnosis. A gastric trichobezoar was ultimately identified on esophagogastroduodenoscopy (EGD). While uncommon, trichobezoars should be considered in the differential diagnosis of pediatric patients, especially females presenting with nonspecific symptoms. Without obvious alopecia or a known psychiatric history, diagnosis is often delayed due to symptoms overlapping with other conditions, and EGD evaluation should be considered.

## 1 Introduction

Trichobezoar, derived from the Greek word *trich,* meaning hair, and *bezoar,* meaning indigestible ball, is formed by the accumulation of ingested human hair within the gastric lumen. Its formation results from the hair's resistance to peristalsis and digestion (1). Continuous ingestion of hair, often mixed with food particles leads to an increase in volume, creating gastric outlet obstruction, abdominal pain, distension, and vomiting (1). Trichobezoars are commonly seen in young females and are associated with trichotillomania and trichophagia (2). In rare cases, the trichobezoar can extend from the stomach into the jejunum or beyond, a presentation known as “Rapunzel Syndrome”. This form can lead to more severe complications due to mechanical irritation and ulceration of the intestinal mucosa (1, 2).

Diagnosis can be established through endoscopy or imaging. Here, we present a case of gastric trichobezoar that closely mimicked SMA across multiple imaging modalities, leading to delayed diagnosis. It was only because of increased clinical concern for persistent patient's symptoms, that endoscopy was envisioned and performed.

## 2 Case description

A 7-year-old previously healthy girl presented with a 3-week history of intermittent upper abdominal pain, early satiety, and 4.5-kilogram weight loss. The food intake was poor, though fluid intake was adequate. There was no vomiting, diarrhea, or altered bowel habits.

On examination, she was afebrile with normal vital signs for age. There were normoactive bowel sounds and generalized abdominal tenderness without distention.

### 2.1 Investigations

**Figure 1:**
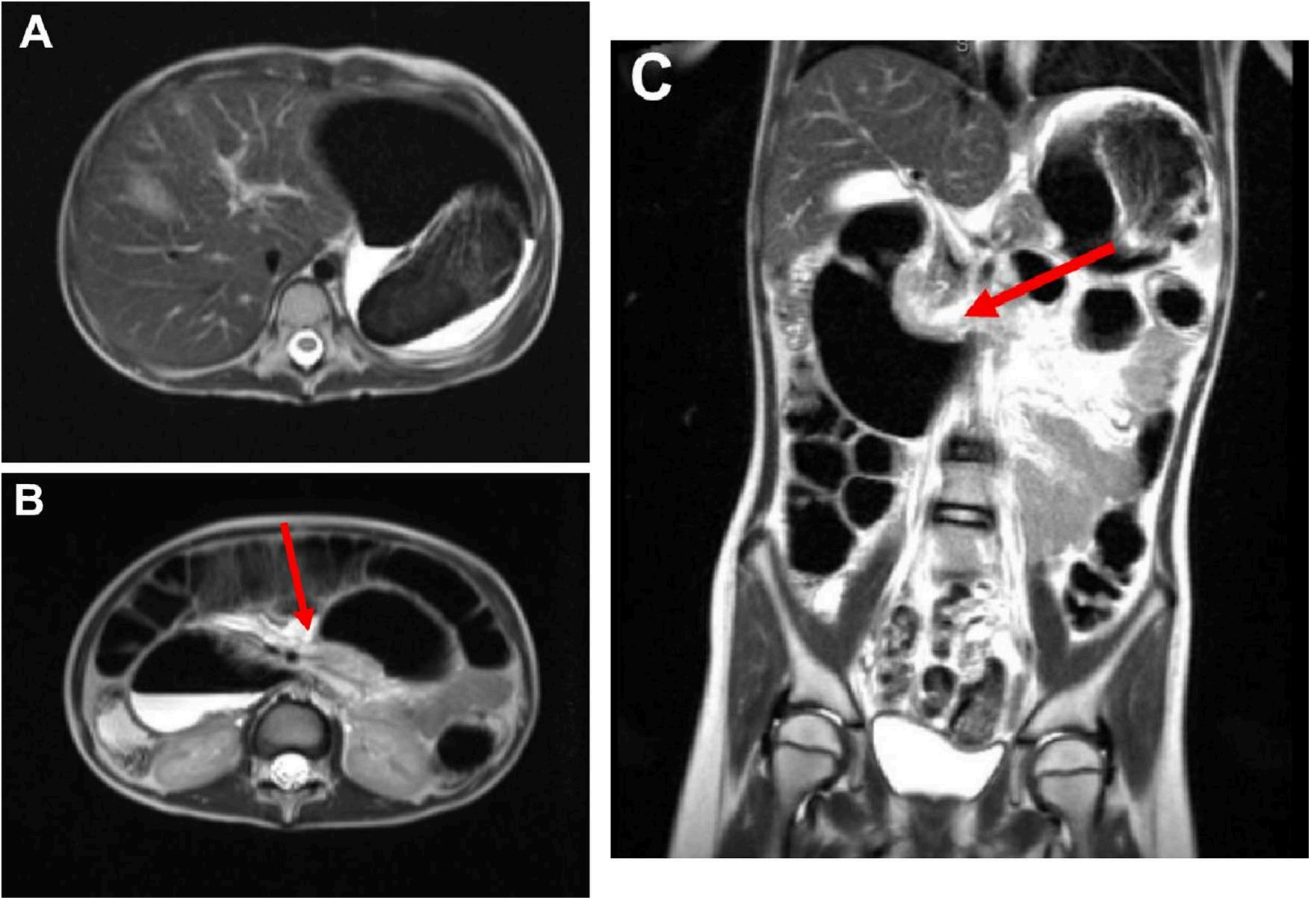
MRI of the abdomen and pelvis. **(A)** An axial view of a T2 low signal mass-like structure in the gastric fundus. **(B)** Dilation of the second and proximal third portions of the duodenum with severe narrowing at the level of the SMA. **(C)** A coronal view showing duodenal dilation with narrowing in the region of the SMA.

Our patient's complete blood count was notable for normal white blood cell count (11.6 × 10^3^ cells/*μ*L), normal hemoglobin (13.1 g/dL), and mild thrombocytosis (499 × 10^3^ platelets per unit). Lipase was mildly elevated (107 U/L), and iron studies revealed iron deficiency (Fe 17 ug/dL, total iron-binding capacity 345 mcg/dL, iron saturation 5%). Abdominal radiography and ultrasound were normal. Given the patient's persistent symptoms and degree of weight loss, MRI of the abdomen and pelvis was pursued to further evaluate for organic pathology while avoiding ionizing radiation. MRI demonstrated significant gastric and proximal duodenal distention with apparent narrowing at the level of the SMA, findings that were interpreted as suggestive of SMA syndrome (Figure 1).

**Figure 2:**
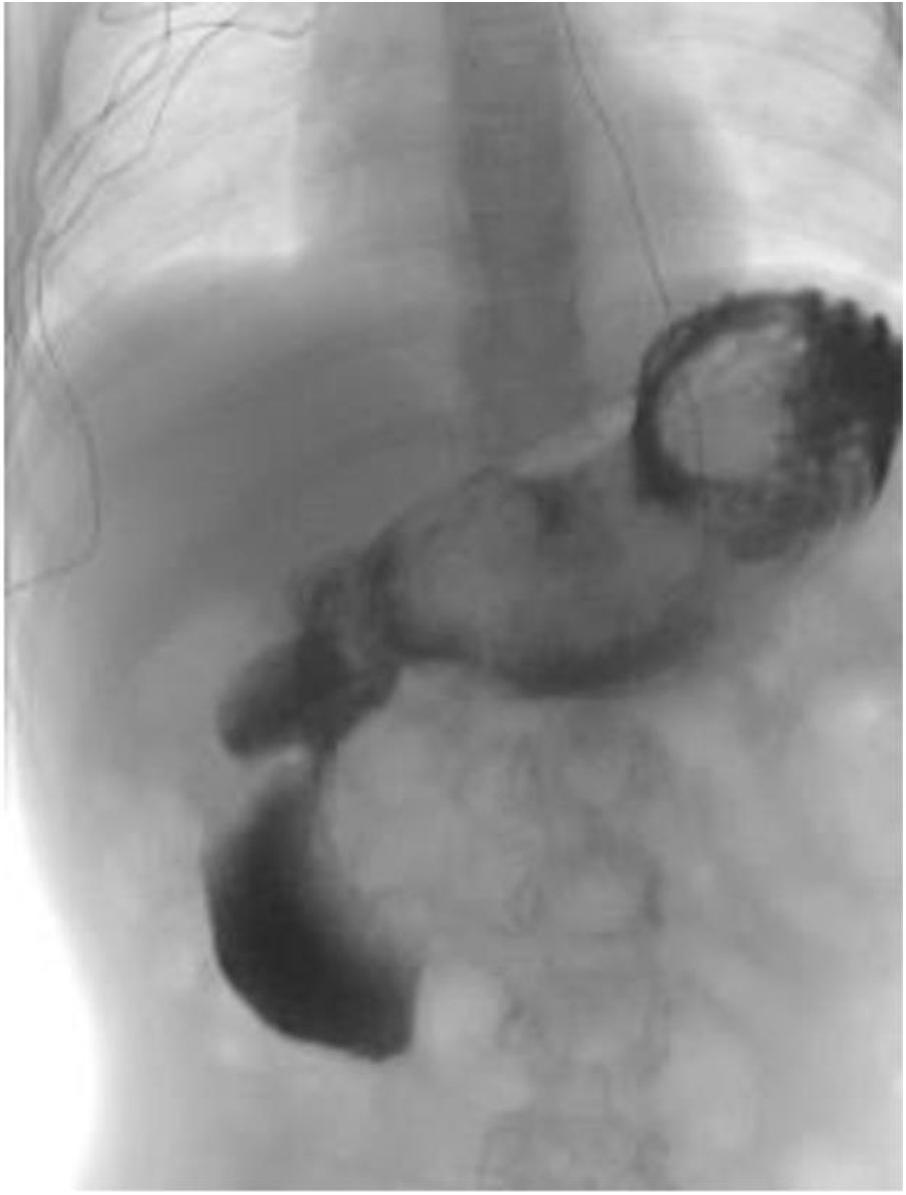
UGI series. Dilation of second portion of duodenum with delayed emptying.

To further assess for functional obstruction, an UGI series was obtained. This revealed delayed gastric emptying, dilatation of the second portion of the duodenum, and improvement with positional changes, findings that reinforced the working diagnosis of SMA syndrome (Figure 2).

### 2.2 Therapeutic intervention

**Figure 3:**
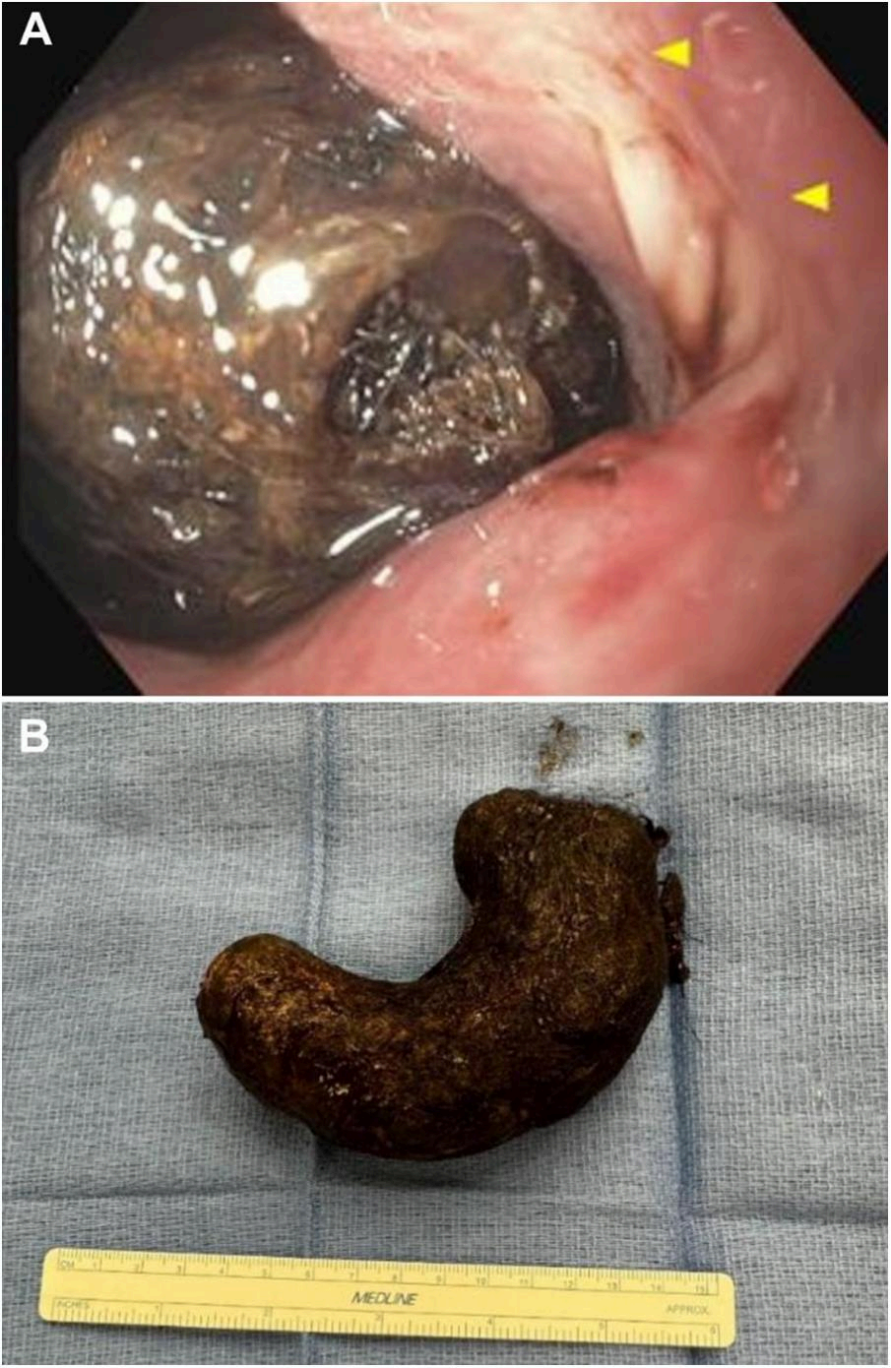
EGD findings and post-laparoscopy with gastrotomy. **(A)** Endoscopic visualization of a large trichobezoar with adjacent gastric body ulceration. **(B)** 10.0 × 3.2 × 3.0 cm intact gastric trichobezoar.

The presence of a persistent gastric filling defect raised concern for an intraluminal process. Given the discrepancy between imaging findings and symptom severity, an EGD was performed, which revealed a large trichobezoar with surrounding ulceration in the gastric body (Figure 3A). Due to the size and density of the trichobezoar, endoscopic removal was not attempted and surgical removal was recommended. Pediatric surgery performed a laparoscopic-assisted gastrostomy with an *en bloc* extraction of an intact gastric trichobezoar measuring 10.0 × 3.2 × 3.0 cm (Figure 3B).

### 2.3 Outcome and follow-up

The postoperative course was uncomplicated, and the patient was discharged home on oral feeds and a proton-pump inhibitor for treatment of the gastric ulcer. Upon further discussion with parents following the trichobezoar diagnosis, the patient was noted to have a habit of sucking and ingesting her hair during periods of anxiety. Outpatient behavioral health follow up was arranged to address trichotillomania and trichophagia, and the patient's parents were counseled on strategies to monitor and reduce recurrence risk. After one-week follow-up, the patient had appropriate oral intake, regained 2.3 kg, and had no recurrence of abdominal symptoms. No further hair-ingesting behaviors were reported at that time.

## 3 Discussion

Trichobezoar is rare and occurs in less than 1% of the pediatric population, predominantly affecting females (3). In children, an organic etiology should be suspected when there is chronic weight loss, recurrent vomiting, or anorexia (4).

Imaging is often the initial step in the evaluation of pediatric patients with abdominal pain and weight loss; however, its diagnostic utility for trichobezoars is variable. Plain radiographs may demonstrate nonspecific findings such as gastric distention or mottled gas patterns but provide limited information regarding intraluminal masses (4). Only 10%–18% of bezoars are identified using radiography alone (5, 6). In this case, abdominal radiographs revealed only an air-filled colon and moderate stool burden without evidence of obstruction. Ultrasound can suggest the presence of bezoar through echogenic intraluminal masses with posterior acoustic shadowing, yet its sensitivity is limited by trapped air and food debris (6). As described by S. McCracken et al., a “clean” acoustic shadow may indicate foreign material rather than gas, but these findings are not consistently present. In our patient, both radiography and ultrasound were non-diagnostic, which contributed to diagnostic uncertainty in our patient.

Contrast-enhanced computed tomography (CT) is the preferred imaging study for suspected trichobezoars, with reported high sensitivities up to 90% and specificities up to 60% (7). Highly diagnostic findings may reveal hypodense, heterogenous gastric mass with a mesh-like appearance or a well-defined heterogenous intraluminal mass at the transition zone (7). However, due to concerns regarding ionizing radiation in a young child, CT imaging was deferred in this case.

MRI, while advantageous in avoiding radiation exposure, has limited evidence supporting its accuracy in detecting bezoars (8). Large gastric trichobezoars may appear as low-signal intensity structures across multiple sequences and can be mistaken for intragastric air (9). In our patient, MRI demonstrated non-specific gastric and proximal duodenal dilation without radiological evidence of discrete intraluminal mass or abnormal gastric contents, findings that were suggestive of SMA syndrome.

UGI series can reveal filling defects and dilation proximal to an area of narrowing (10, 11). In this case, UGI series demonstrated delayed gastric emptying and duodenal dilation with positional improvement. These findings, along with our patient's rapid weight loss, initially raised concern for SMA syndrome, a rare cause of gastric outlet obstruction caused by a loss of mesenteric fat pad and compression of the third portion of the duodenum (12, 13). SMA syndrome primarily affects young females and shares overlapping symptoms with trichobezoar including nausea, vomiting, epigastric pain, and early satiety. The diagnostic anchoring toward SMA syndrome, in this case, was influenced by clinical and imaging features rather than definitive evidence of extrinsic vascular compression. In retrospect, the persistent gastric filling defect seen on UGI could be attributed to intraluminal material. A key distinction lies in the mechanism of obstruction: extrinsic compression in SMA syndrome vs. intraluminal obstruction in trichobezoar.

This case illustrates how reliance on imaging alone, particularly when findings are interpreted in isolation, can delay consideration of alternative diagnoses. In such scenarios, EGD plays a critical role in differentiating intraluminal from extrinsic causes of obstruction.

EGD remains the gold standard for diagnosis of trichobezoar, as it allows for direct visualization of intraluminal masses, assessment of mucosal injury, and potential therapeutic interventions in selected cases (2, 11, 13). In our patient, EGD confirmed the presence of a large gastric trichobezoar and revealed adjacent gastric ulceration, accounting for the patient's iron deficiency.

Although our patient's bezoar was confined to the stomach, recognition of “Rapunzel Syndrome” remains important. This rare variant occurs when the trichobezoar extends beyond the pylorus into the small intestine. In such cases, the trailing portion of the hair mass can become embedded with overgrown intestinal mucosa, increasing the risk of complications including mucosal ulceration, gastrointestinal bleeding, bowel ischemia, and perforation. These sequalae may require surgical intervention (1, 3, 11). In contrast, while mucosal injury and gastric ulcers can also occur with longstanding gastric bezoars, the stomach lacks the same degree of mucosal incorporation seen in intestinal extensions.

Beyond acute management, long-term outcomes depend on addressing underlying behavioral and psychiatric factors. Trichobezoars are strongly associated with trichotillomania and trichophagia; however, these behaviors may not be readily apparent at initial presentation. In this case, focused history obtained after diagnosis revealed that the patient had a habit of sucking and ingesting her hair during periods of anxiety. This behavior was not initially volunteered by the patient or her parents and was only identified after the trichobezoar was discovered. The absence of overt alopecia or a known psychiatric contributed to delayed clinical suspicion. Consequently, multidisciplinary follow-up involving pediatric surgery, gastroenterology, and behavioral health services is essential to reduce the risk of recurrence (1, 3).

In conclusion, trichobezoars should be considered in pediatric patients with unexplained GI symptoms, weight loss, or alopecia. While extensive imaging can provide valuable diagnostic clues, they may be misleading or non-diagnostic in cases of intraluminal obstruction. Early consideration of endoscopy is critical when symptoms persist despite equivocal imaging Awareness of these diagnostic pitfalls and a multi-disciplinary approach are key to timely diagnosis, prevention of complications, and reduction of recurrence risk.

## Data Availability

The raw data supporting the conclusions of this article will be made available by the authors, without undue reservation.
